# Combined effect of salt stress and *Xanthomonas axonopodis* pv *citri* on citrus (*Citrus aurantifolia*)

**DOI:** 10.1016/j.heliyon.2020.e03403

**Published:** 2020-02-12

**Authors:** Shahran Ahmed Nayem, M. Salahuddin M. Chowdhury, Nazneen Sultana, Gazi Ziaul Haque Masum, Md. Shahedur Rahman, Mohammad Abu Hena Mostofa Jamal

**Affiliations:** a Dept of Plant Pathology, Sher-e-Bangla Agricultural University, Dhaka, Bangladesh; b Dept. of Biotechnology and Genetic Engineering, Islamic University, Kushtia, Bangladesh; c Dept. of Genetic Engineering and Biotechnology, JUST, Jessore, Bangladesh; d Department of Horticultural Science Technical University of Munich, Freising, Germany

**Keywords:** Agriculture, Plant biology, Salt stress, Chlorophyll content, Net assimilation rate, Intercellular CO2 concentration, Stomatal conductance, Transpiration rate, Citrus canker

## Abstract

*Xanthomonas axonopodis* pv *citri* (Xac) and salt stress are two crucial hindrances to citrus production. The effect of continuous salt application and Xac infection in citrus has been investigated. Citrus plants were exposed to salt stress by irrigating with 50 mM, 100 mM, 150 mM, and 200 mM NaCl solution on weekly basis and challenged by Xac. Salt stress affected the defense response of Citrus plants to Xac and therefore lesion diameter and disease severity were gradually increased at higher salt concentration. Meanwhile, accumulation of Na^+^ and Cl^−^ in the leaves were also increased with the increase of salt concentration. Besides, physiological performance (PP) of plants was estimated based on the parameters such as net assimilation rate, chlorophyll content, stomatal conductance, transpiration rate and intercellular CO_2_ concentration. The PP of sole Xac treated plants was gradually increased and maintained up to end of the experiment, whereas plants treated with Xac+50 mM and Xac+100 mM NaCl showed the highest PP up to 30 days after inoculation and then decreased. However, the PP of Xac+150 mM and Xac+200 mM NaCl treated plants gradually decreased till the end of experiment. Similarly, the PP of 200 mM NaCl treated plants declined continuously. Interestingly, the PP in 50 mM and 100 mM NaCl treated plants was higher initially and then decreased at 30 DAI to 40 DAI. This study revealed that citrus canker disease development was enhanced by salt stress. In addition, the physiological performance of the plants was enhanced by Xac and Xac + moderate salt stress but then demolished under severe salt stress.

## 1 Introduction

Plants need to confront with a wide sort of stress to survive in natural condition. Salinity, an abiotic stress and pathogen, a biotic stress are largely two prominent factors that plants need to deal with for survival. Salt stress is being a worldwide threat for agriculture day by day. Salinity is accumulation of soluble salts in cellular level (Levy and Syvertsen, 2004) which causes adverse morphological, physiological, and biochemical effects in different organs of citrus plants through an increased concentration of sodium and chloride ions (Boman, 1994; Camara-Zapata et al., 2004; Raveh and Levy, 2005). Salt stress could increase the prevalence of many citrus diseases (Afek and Sztejnberg, 1993; Dann et al., 1998), but no information is available about the effect of salinity on citrus canker disease. *Xanthomonas axonopodis* pv. *Citr* (Xac), incitant of citrus canker is a century old biotrophic pivotal bacteria. The prevalence of this universal bacteria is highest in arid and semi-arid region due to environmental facilities. Xac is the only known bacteria yet that can produce XacPNP gene which is mobile protein signaling molecules and secreted into the apoplast to regulate plant homeostasis (Wang et al., 2011). This bacterium can encode (Plant Natriuretic Peptide) PNP like protein which has sequence similarity and domain identity with plant PNPs (Gottig et al., 2008). However, plant showed unwonted response to a combination of two stresses (Mittler, 2006; Atkinson and Urwin, 2012). Although, the effect of single stressor like salinity, drought and pathogen infection have been copiously investigated, very meager information is available about how a combination of different stresses affects plants. Salt stress can either reduce or enhance disease severity. Salt stress enhanced resistance of barley (*Hordeum vulgare*) against barley powdery mildew (*Blumeria graminis* f.sp. *hordei*) (Wiese et al., 2004). Likewise, in cucumber, salt stress increased disease severity and induced susceptibility to *Pseudomonas syringae* pv. *lachrymans* (Chojak et al., 2012). Furthermore, salt stress inhibits defense mechanism of citrus plant against *Phytophthora* pathogen (Blacker and Mcdonald, 1986). Again, salinity have negative effect on citrus physiology (Balal et al., 2012). It affects net assimilation rate through reducing stomatal conductance and water uptake which led to an increase of intercellular CO_2_ concentration. The high net assimilation rate indicates lower intercellular CO_2_ in mesophyll tissue. To understand the combined effect of a biotic and abiotic stress on citrus plant we determined the impact of simultaneously applied salt stress and Xac infection on citrus plant.

## 2 Materials and methods

### 2.1 Isolation, culture and identification of causal organism

*Xanthomonas axonopodis* pv *citri* the causal organism of citrus canker was isolated from infected lemon leaf by dilution plate techniques according to Goszczynska and Serfontein (1998). Bacterial colony was purified by culturing on SX media (semi selective media for *Xanthomonas*) and maintained in NB (Nutrient Broth) containing Beef extract 3 gL^-1^ and peptone 5 gL^-1^ at 28 °C for 48 h at shaker incubator and then cultured in NA (Nutrient Agar) media containing Beef extract 3 gL^-1^, Peptone 5 gL^-1^ and agar 15 gL^-1^ and subculture at one-month interval. The citrus canker bacterium was identified by gram's staining reaction (Gerhardt, 1981), oxidase test (Kovacs, 1956), potassium hydroxide (3% KOH) test (Suslow et al., 1982), catalase test, starch hydrolysis test, Tween 80 lypolysis test (Schaad, 1992) esculin hydrolysis test, milk proteolysis test, Gelatin liquefaction test, citrate utilization test, and Pathogenicity Test (Lin et al., 2008).

### 2.2 Cultivation of planting material

One-year old, vigor and healthy lemon (*Citrus aurantifolia*) seedlings were collected from Krishibid nursery Agargaon, Dhaka. The seedlings were grown (one plant/pot) in twenty-inch earthen pot. The potting media was prepared using sterile loamy soil and sand in 2:1 ratio. Potted seedlings were irrigated three times in a week with distilled water. The plants were not supplied with additional nutrients.

### 2.3 Preparation and application of salt solution

2.9 g, 5.8 g, 8.7 g and 11.6 g NaCl were weighted and diluted to 1000 ml sterilized distilled water separately to obtain 50 mM, 100 mM, 150 mM and 200 mM respectively (Banuls and Millo, 1992). The plants were irrigated with salt solution two times during the whole experiments using 500 ml solution per pot. The plants were first irrigated with salt solution after one month of transplanting in pot and the second salt irrigation was done three weeks after the first irrigation. Thus, each plant got total 2.90 g salt for 50 mM treatment, 5.8 g for 100 mM treatment, 8.7 g for 150 mM treatment and 11.6 g for 200 mM treatment up to the end of experiment.

### 2.4 Inoculums preparation and inoculation

The inoculums were grown in Nutrient broth (NB) for 24 h in 30 °C temperature. The inoculums were injected by syringe infiltration into the abaxial surface of the leaf with an OD value 0.5_650nm_ (approximately 10^8^ cfu/ml). Each leaf was inoculated with 0.5 ml bacterial solution. Seven days after salt treatment, the plants were inoculated with bacteria.

### 2.5 Experimental design and treatments

The experiment was done with 9 treatments and three replications in each treatment in complete randomized design (CRD). The treatments were Healthy plants/Control 1, Xac + water treated plants/Control 2, 50 mM NaCl, 100 mM NaCl, 200 mM NaCl, Xac +50 mM NaCl, Xac+100 mM NaCl, Xac +150 mM NaCl and Xac +200 mM NaCl.

### 2.6 Extraction and estimation of Na^+^ and Cl^−^ ions from leaf

Sodium was extracted by Di-acid mixer method (Woldring, 1953). The Di-acid mixer was prepared by adding 60% HClO_4_ to conc. HNO_3_ in a 2:1 ratio. One-gram oven dried leaves sample was taken in 250 ml conical flask. 20 ml Di-acid mixture was added to it. It was then heated at 200 °C until white fume evolved and after that it was cooled, and 20–30 ml distilled water was added. This solution was then filtered and a final volume of 100 ml was prepared by adding distill water. The concentration of sodium in the test sample was determined by flame emission spectrophotometer from the standard curve prepared by standard NaCl solution and expressed as mg g^−1^ of fresh sample materials. Leaf chloride amount was calculated as precipitation of silver chloride by titration with silver nitrate according to Johnson and Ulrich (1959) and the amount was expressed as mg g^−1^ of fresh sample materials.

### 2.7 Data collection and analysis

Data on lesion diameter, disease severity, Chlorophyll content, Net CO_2_ assimilation rate, Stomatal conductance, Transpiration rate and Intercellular CO_2_ concentration were recorded at 10, 20, 30 and 40 days after inoculation. Initially, the 0–5 grade disease severity scale (Rai and Mamatha, 2005) was used to record the disease severity. The grades were, 0 = 0 % infection, 1 = 1% infection, 2 = 1–10% infection, 3 = 10–20% infection, 4 = 20–40% infection and 5 = 40–100% infection. The final disease severity was counted according to the following formula

$$\text{Percent Disease Index (PDI)}\;=\;\frac{Sum\;of\;individual\;disease\;rating}{Total\;number\;of\;leaf\;examined\;\times Maximum\;number\;of\;grade}\times 100$$

The average Chlorophyll content was recorded from five leaves per plant by using “S-PAD” meter (KONICA MINOLTA INC, Osaka, Japan). Net CO_2_ assimilation rate, Stomatal conductance, Transpiration rate and Intercellular CO_2_ concentration were recorded from five leaves per plant by using LCpro + portable infrared gas analyzer (ADC Bioscientific Ltd., Hoddesdon, UK) under humidity and ambient CO_2_ machine. 1000 molm^−2^s^−1^photon flux density light was provided by a photosynthetically active radiation lamp. All measurements were performed at 12 p.m. Data comparison was done by one-way analysis of variance (ANOVA) and mean difference through LSD at P = 0.05 using STATISTIX 10 software (Analytical software, USA). The data points are the mean of three independent experiment (n = 3) and three leaves per plant and three plants per treatment were examined in each experiment. Standard deviation and correlation were calculated by using Microsoft excel software.

## 3 Results

### 3.1 Identification of causal organism

The isolated causal organism was identified as *Xanthomonas axonopodis* pv. *Citri* based on basis of morphological, biochemical characters. The bacteria showed light yellow to slightly blue, flattened, small, growth on SX agar plate and yellow, convex, mucoid growth on NA plates after 48 h of incubation at 30 ± 1 °C. The bacterium showed red color in gram staining test and was rod shape. In addition, the bacterium produced a mucoid thread when lifted with the loop in KOH test. Besides, it showed catalase activity, bubbles were formed after adding 3% H_2_O_2_. The bacterium formed dark purple color on oxidase disk, showed β-glycosidase activity in esculin hydrolysis test. Again, the bacterium produced esterase enzyme in tween 80 lypolysis test, liquefied the gelatin, proteolyzed the milk, utilized the citrate, hydrolyzed the starch (Table 1). Besides, the bacterium showed positive result in pathogenicity test.

**Table 1:** Biochemical test result of pathogenic bacteria of citrus canker.

Biochemical tests	Results
Oxidase test	Negative
Catalase test	Positive
KOH solubility test	Positive
Gelatine liquefaction test	Positive
Starch hydrolysis test	Positive
Tween 80 typolysis	Positive
Milk protolysis	Positive
Citrate utilization test	Positive
Aesculin hydrolysis	Positive

### 3.2 Endogenous Na^+^ and Cl^−^ level in leaves of salinized and non-salinized plants

**Figure 1:**
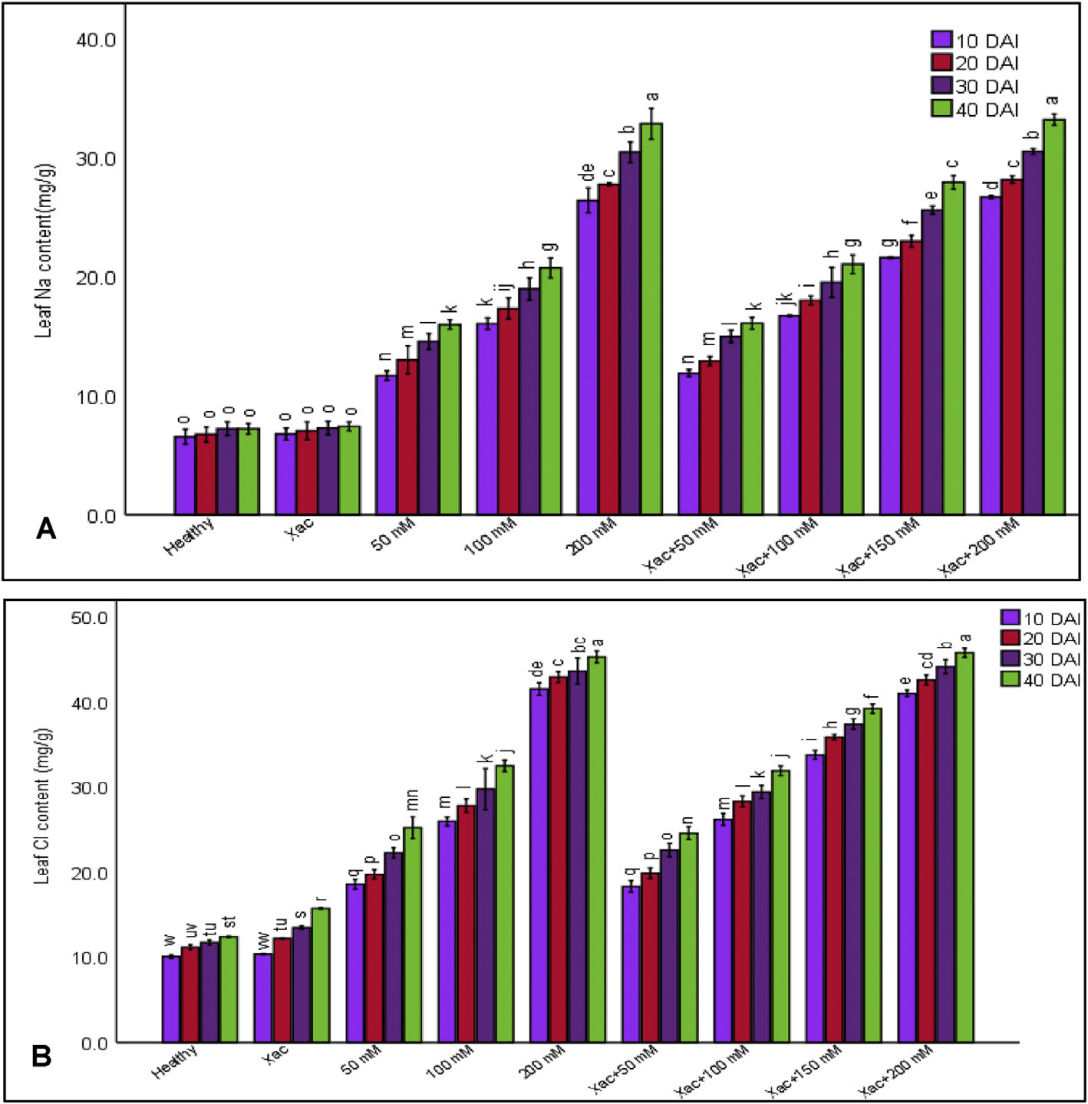
Comprison of leaf Na and Cl content in healthy, Xac and Xac + Salt treated citrus plants. (A) Leaf Na content (B) Leaf Cl content. Data indicates the mean value of treatments from 3 individual experiments with 3 replications in each experiment (n = 9). Different lowercase letters above the bar indicate significant at P = 0.05.

Amount of Na^+^ and Cl^−^ ions was assessed with a view to perceiving that whether the NaCl applied in the root zone were accumulated in the leaf or not. The accumulation of Na^+^ and Cl^-^was notably significant in salinized plants than non-salinized plants at 10 DAI, 20 DAI, 30 DAI and 40 DAI respectively (Figure 1). Among the treatments, 200 mM NaCl treated plants had fourfold highest leaf Na^+^ content (33.23 mg g^−1^) at 40 DAI, compared with non-salinized plants Control-1 (7.60 mg g^−1^) and Control-2 (7.63 mg g^−1^). Besides, 50 mM, 100 mM and 150 mM NaCl treated plants had a moderate and statistically significant leaf Na^+^ content (16.10 mg g^−1^, 21.06 mg g^−1^ and 27.96 mg g^−1^) but the former two were statistically similar up to 30 DAI and then it was significant. In addition, leaf Cl^−^ content measured at 40 DAI was enhanced over three times from 15.63 mg g^−1^ to 45.83 mg g^−1^ in control to 200 mM NaCl treated plants. Significant and moderate leaf Cl^−^ content (24.63 mg g^−1^, 31.96 mg g^−1^ and 39.23 mg g^−1^) was estimated on the leaf derived from 50 mM, 100 mM and 150 mM NaCl treated plants. The data indicate that application of NaCl in rhizosphere led to an increase of Na^+^ and Cl^−^ ions in leaf.

### 3.3 NaCl treatment enhance canker development in citrus plant

**Figure 2:**
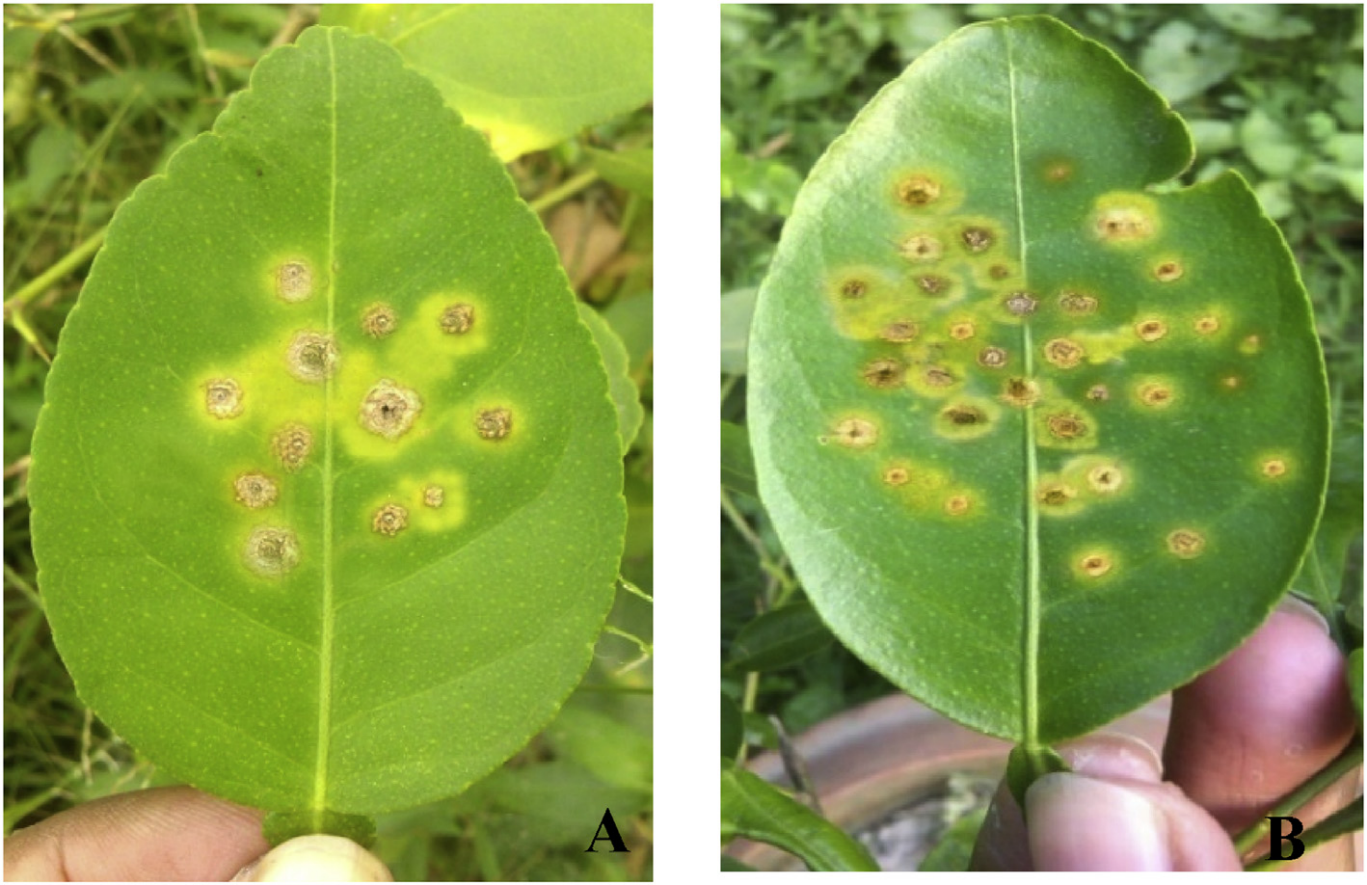
Disease symptoms at 40 days after inoculation (A) Only Xac treated plant (B) Xac+ 200 mM NaCl treated plants.

Canker development was significantly increased with the increase of salt concentration and exposure period of plants into salt solution. When salt treated plants challenged with Xac, they expressed symptoms at 10 days after inoculation (DAI). Conversely, non-salinized plants (Control-2) didn't express symptom at 10 DAI (Table 2). However, plants varied significantly in the disease severity (DS), 10.93 % and 10.53 % for the 200 mM NaCl and 150 mM NaCl treatment respectively and 0.00 % for the control 2. Meanwhile, moderate and statistically significant DS, 3.20 % and 5.93 % were recorded at 100 mM NaCl and 150 mM NaCl treatment respectively. Again, at 20 DAI non-salinized plants expressed symptoms, whereas DS significantly increased at 200 mM NaCl treated plants (24.67%) compared to Control-2 (8.33%). Though the DS of both salts treated, and non-treated plants increased by degrees prior to the end of experiment, 200 mM NaCl treated plants showed the highest severity (34.60%, 52.33%) compared to Control-2 (14.07%, 24.93%) at 30 DAI and 40 DAI. However, 50 mM and 100 mM salt treated plants showed a moderate DS at 30 DAI and 40 DAI. Furthermore, lesion diameter (LD) also augmented progressively till the last day of experiment. At 40 DAI, 200 mM NaCl treated plants showed the largest LD (10.33 mm^2^) which was more intense than Control-2 (3.33 mm^2^) (Table 3 and Figure 2). At the same time 50 mM and 100 mM NaCl treated plants had the moderate and statistically significant LD (7.60 mm^2^ and 8.00 mm^2^). The above findings indicate that NaCl treatments upturned canker disease development.

**Table 2:** Disease severity of citrus canker disease under different salt concentration.

Treatments	Disease severity (%)			
	10 DAI	20 DAI	30 DAI	40 DAI
Healthy plant (Control-1)	0.00 d	0.00 e	0.00 e	0.00 e
Xac inoculated plant (Control-2)	0.00 d	8.33 d	14.07 d	24.93 d
Xac +50 mM NaCl	3.20 c	11.33 c	21.47 c	32.60 c
Xac +100 mM NaCl	5.93 b	17.80 b	28.00 b	40.80 b
Xac +150 mM NaCl	10.53 a	24.67 a	34.47 a	51.20 a
Xac +200 mM NaCl	10.93 a	23.60 a	34.60 a	52.33 a
LSD (0.05)	0.97	1.71	2.74	2.50
CV (%)	8.77	5.50	5.69	3.42
Level of Significance	*	*	*	*

*^- significant at P=0.05. Data indicates the mean value of treatments from 3 individual experiments with 3 replications in each experiment (n=9). Different lowercase letters beside the mean value indicate significant at P= 0.05^.

**Table 3:** Lesion diameter of citrus canker disease under different salt concentration.

Treatments	Lesion diameter (mm^2^)			
	10 DAI	20 DAI	30 DAI	40 DAI
Healthy plant (Control-1)	0.00 d	0.00 d	0.00 d	0.00 c
Xac inoculated plant (Control-2)	0.00 c	2.60 b	5.60 a	9.46 a
Xac +50 mM NaCl	0.69 b	1.06 c	1.66 c	7.60 b
Xac +100 mM NaCl	1.03 b	2.40 b	3.00 b	8.00 b
Xac +150 mM NaCl	1.93 a	4.40 a	6.26 a	9.33 a
Xac +200 mM NaCl	2.13 a	4.73 a	6.13 a	10.33 a
LSD (0.05)	0.34	0.63	0.82	1.27
CV (%)	16.14	11.55	10.06	9.45
Level of Significance	*	*	*	*

*^- significant at P=0.05. Data indicates the mean value of treatments from 3 individual experiments with 3 replications in each experiment (n=9). Different lowercase letters beside the mean value indicate significant at P= 0.05.^

### 3.4 Determination of correlation coefficient and regression equation

**Figure 3:**
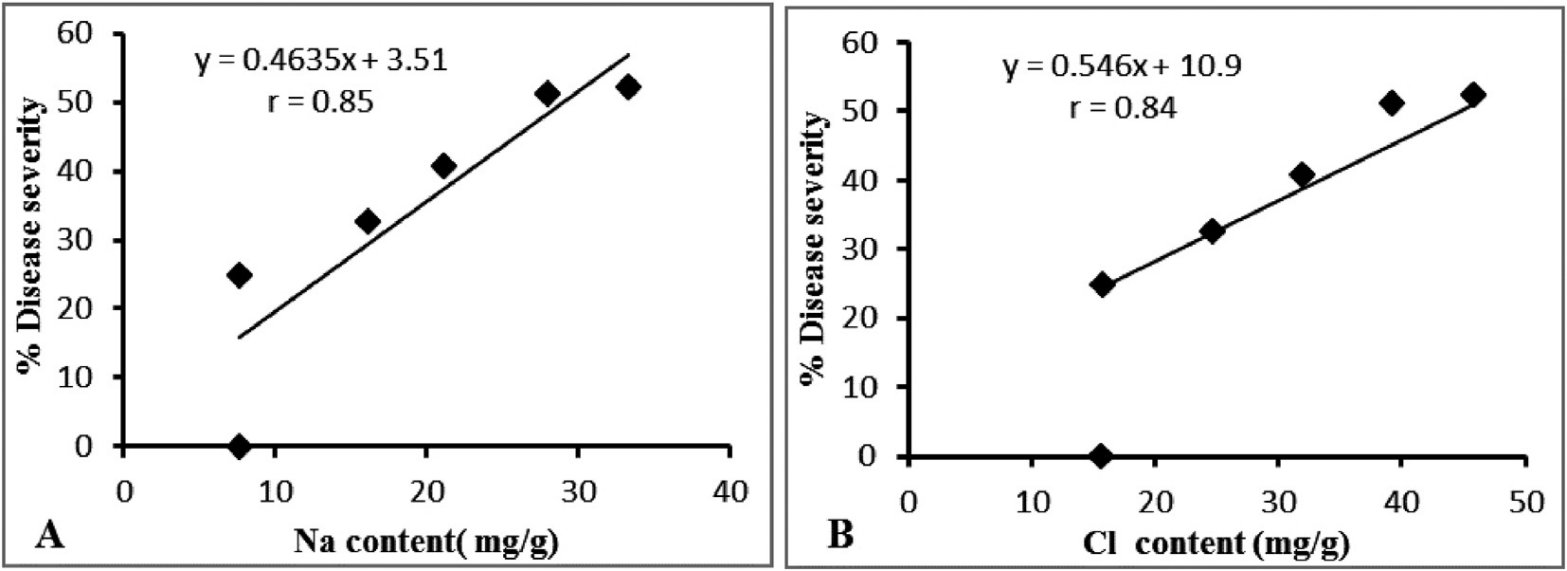
Correlation between (A) Disease severity and leaf Na content (B) Disease severity and Leaf Cl content at 40 days after inoculation. Data indicates the mean value of treatments from 3 individual experiments with 3 replications in each experiment (n = 9)The data is significant at P = 0.05.

The plants treated with different concentration of NaCl and Xac showed boosted disease severity with increased Na^+^ and Cl^−^ ions. A positive correlation was found between disease severity and Na^+^ level (Figure 3A). Similarly, the correlation between disease severity and Cl^−^ level was also positive (Figure 3 B). The regression equation also demonstrated the positive correlation between disease severity and Na^+^ level as well as severity and Cl^−^ level.

### 3.5 Physiological performance of plant under the combination of pathogen and salt stress

**Figure 4:**
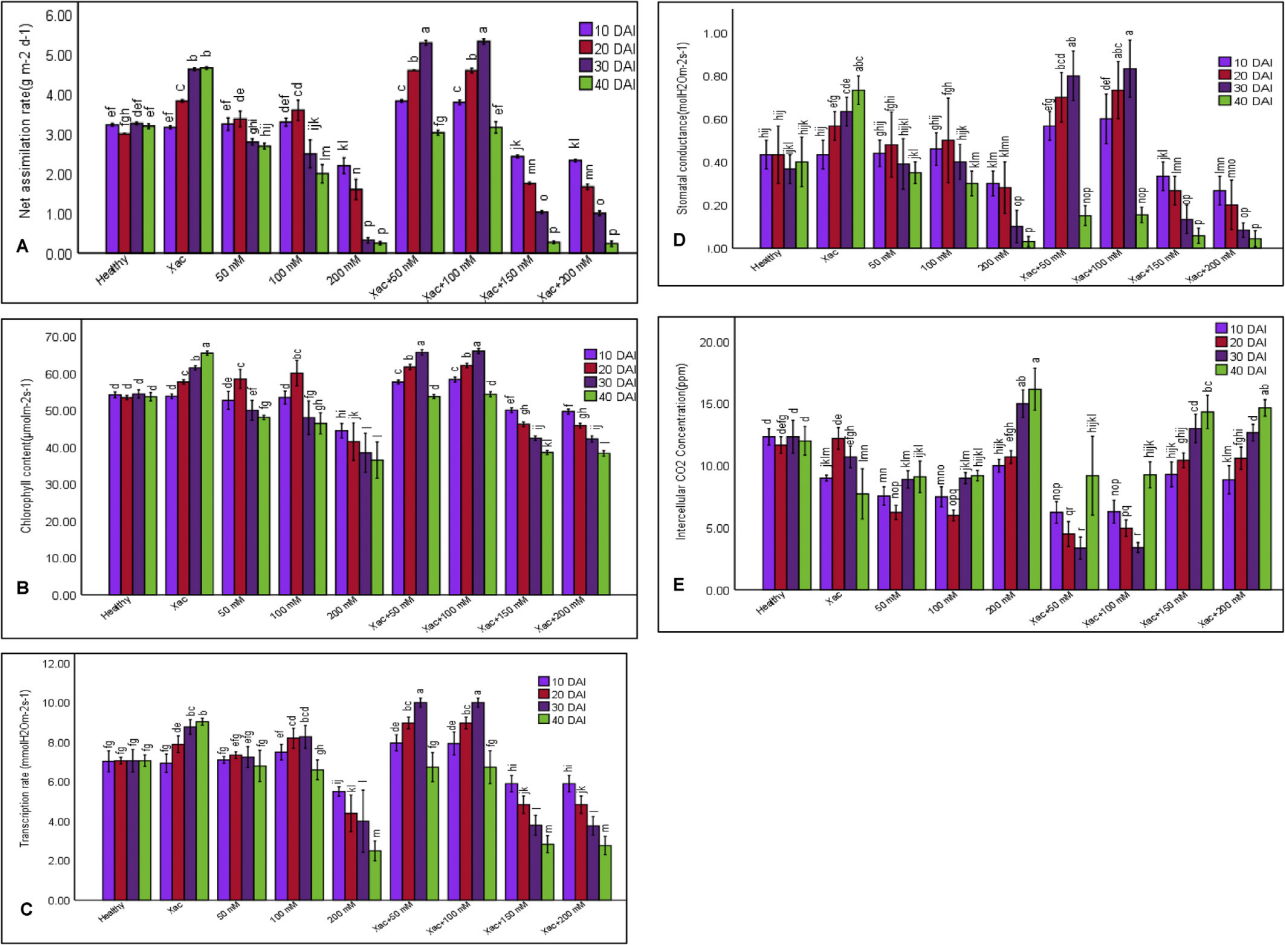
Comparison of physiological parameters of healthy plants, Xac inoculated plants and (Xac + salt) treated plants of citrus. (A) Net Assimilation Rate (B) Chlorophyll Content (C) Transpiration Rate (D) Stomatal Conductance (E) Intercellular CO_2_ Concentration. Data indicates the mean value of treatments from 3 individual experiments with 3 replications in each experiment (n = 9). Different lowercase letters above the bar indicate significant at P = 0.05.

Physiological parameters viz: Net assimilation rate (NAR), Chlorophyll content (CC), Intercellular CO_2_ concentration (ICC), Stomatal conductance (SC) and Transpiration rate (TR) were examined to perceive that either the physiological performance (PP) of the plants was modulated by the combined effect of pathogenic and salt stress or not. NAR, CC, SC and TR were increased from 10 DAI to 30 DAI and then abated at 40 DAI in Xac+50 mM and Xac+100 mM NaCl treated plants (Figure 4, A-D). Again, NAR, CC, SC and TR were increased from 10 DAI to 20 DAI in 50 mM and 100 mM NaCl treated plants and then decreased at 30 and 40 DAI. Besides, 200 mM NaCl, Xac+150 mM and Xac+200 mM NaCl treated plants showed a gradual decrease up to 40 DAI, whereas Xac treated plants showed a gradual increase. Besides, CC and TR remained stable in healthy plants from 10 DAI to 40 DAI. On the other hand, ICC, NAR and SC showed fluctuation (Figure 4, E).

At 40 DAI, the highest NAR, CC, SC and TR (4.66 g m^−2^ d^−1^, 65.53 μmolm^−2^s^−1^, 0.73 molH_2_Om^−2^s^−1^ and 9.03 mmolH_2_Om^−2^s^−1^) were recorded in Xac treated plants, conversely 200 mM NaCl treated plants showed the lowest NAR, CC, SC and TR (0.25 g m^−2^ d^−1^, 36.50 μmolm^−2^s^−1^, 0.03 molH_2_Om^−2^s^−1^ and 2.5 mmolH_2_Om^−2^s^−1^). However, 200 mM NaCl treated plants had the highest ICC (16.16 ppm) and Xac treated plants had the lowest ICC (7.73 ppm). At 30 DAI, the maximum NAR, CC, SC and TR (5.33 g m^−2^ d^−1^, 66.10 μmolm^−2^s^−1^, 0.83 molH_2_Om^−2^s^−1^ and 10 mmolH_2_Om^−2^s^−1^) were estimated in Xac+100 mM NaCl treated plants which was statistically similar with Xac+50 mM NaCl treated plants but 200 mM NaCl and Xac+200 mM NaCl treated plants showed the minimum NAR, CC, SC and TR (0.32 gm^−2^d^−1^, 38.50 μmolm^−2^s^−1^, 0.1 molH_2_Om^−2^s^−1^, 4.00 mmolH_2_Om^−2^s^−1^ and 1.00 gm^−2^d^−1^, 42.23 μmolm^−2^s^−1^, 0.08 molH_2_Om^−2^s^−1^ and 3.76 mmolH_2_Om^−2^s^−1^). Alternatively, 200 mM NaCl treated plants showed the maximum ICC (15 ppm) and Xac+100 mM NaCl treated plants showed minimum ICC (3.40 ppm). Similar pattern of result was observed at 10 DAI and 20 DAI. Above data indicates that plant physiology was increased by the Xac and Xac + moderate salinity whereas, it reduced by severe salinity.

## 4 Discussion

Salt stress, a momentous environmental stress alters the anatomical, physiological and biological processes of plants. Xac is manifested as a hazardous biotic stress for citrus so far. Nonetheless, it is pinpointed for its's positive role to maintain plant physiology (Nembaware et al., 2004). In our study, initially we found that salt treatment enhanced canker disease development which was undoubtedly manifested by the higher disease severity and lesion diameter. This synergistic effect agrees with previous investigations (Blaker, 1986; Afek and Sztejnberg, 1993; Dann et al., 1998). Salinity has positive effect on disease development caused by *Oidium* and *Phytophthora* fungi in tomato plant (Kissoudis et al., 2014). Similarly, salt stress enhance disease susceptibility in tomato against *Pseudomonas* bacteria (Thaler and Bostock, 2004). In the same time, onset of disease symptom was differed between salinized and non-salinized plants. It means salt stress led to a prompt occurrence of canker disease. It is known that disease development at salt stress was increased owing to weak defense mechanism of plants due to hormonal imbalance, down regulation of primary metabolism, knock down of defense response gene and osmotic stress (Chojak et al., 2012). At salt stress, electrolytic leakage was increased indicating cell membrane damage and delay H_2_O_2_ accumulation in leaves that perturbed redox signaling involved in protection response to pathogen. Further, the negative crosstalk between salicylic acid and abscisic acid pathway due to osmotic stress is a possible reason for higher disease development (Mohr and Cahill, 2007). The osmotic stress induces ABA related gene expression which interfere with SA based defense related gene (Hatmi et al., 2018). Afterwards, this study revealed that salinized plants had more Na^+^ and Cl^−^ ions than non-salinized plants in agreement with earlier work (Yassin, 2004; Wu et al., 2009). Augmentation of Na^+^ and Cl^−^ ions accumulation in leaves has been suggested to be seemingly akin to decreased Ca^+^ and K^+^ ions responsible for degradation of cell permeability, division and cell wall thickening (Sudhir and Murthy, 2004; Wu et al., 2009; Silva et al., 2011). Moreover, accumulation of Cl^−^ ion reduces the nitrogen uptake. Due to the imbalance of nitrogen within the plant, enzyme production and activities are impaired (Yassin, 2004). Downturned Ca^+^ and K^+^ ions and nitrogen imbalance is linked with canker development at salt stress. Again, we found that PP of plants was modulated by the combination of biotic (Xac) and abiotic (salt) stress, as manifested by increased NAR, SC, TR, CC and decreased ICC. It was established that salt stress had negative impact on plant physiology due to ionic imbalance, toxicity and osmotic stress (Balal et al., 2012; bib_citation_to_be_resolvedChojak et al., 2012). Alluringly, we observed that plants treated with combination of severe salt (150 mM and 200 mM) and Xac had the lowest PP, which was supported by the highest accumulated leaf Na^+^ and Cl^−^ contents. This excess accumulation of Na^+^ and Cl^−^ ions caused ionic imbalance or osmotic intolerance that yielded lowest PP. It has been suggested that Xac had positive role to maintain plant physiology (Nembaware et al., 2004). In this context, our data revealed that Xac treated plants had an increasing PP with the increase of time after inoculation. It was found that Xac had a power of molecular mimicry wherein it could encode XacPNP gene homologous with plant PNP gene make the condition favorable for it (Wang et al., 2007). This XacPNP gene encodes a protein that regulates photosynthesis, water uptake and stomatal conductance. This gene is activated under stress or poor nutrition since plants could not able to maintain normal physiology (Gottig et al., 2008). Likewise, Xac needed normal physiology of plant cell for its survival and encodes XacPNP gene. Our experiment suggested that plants treated with combination of moderate salt (50 mM and 100 mM) and Xac had the highest PP up to one month after inoculation and then decreased. It has been reported that under salt stress the plant maintains its physiology through ion tolerance and osmotic tolerance and tissue tolerance mechanism (Isayenkov and Maathuis, 2019). Osmotic tolerance involves the reduction of stomatal conductance to preserve water through long distance signaling. On the other hand, the ionic tolerance is maintained by restricting ion efflux from root through activation of several signal cascade. The tissue tolerance covers the compartmentation of ion in vacuoles. The Xac bacterium could contribute to the activation of PNP gene in plant to modulate plant physiology and the PNP gene or its homologs could be involved in above mentioned mechanism to maintain homeostasis within plant under salt stress.

In summary, we found that salt application resulted in escalation of citrus canker disease significantly. Besides, PP of citrus plant was modulated by both salt stress and Xac, wherein Xac bacterium promoted PP till the end of experiment but combination of moderate salt and Xac bacteria elevated PP for a certain period and then downturned. When severe salt treated plants were inoculated with Xac, PP alleviated notably. These finding highlights that although Xac had the ability to regulate physiology of citrus but under severe salt stress this ability demolished. The detected effect of salinity is caused by ionic imbalance and osmotic intolerance of Na^+^ and Cl^−^ ions that compromised plant defense mechanism. We recommend further molecular study on the role of XacPNP gene or its homologs in the involvement of Citrus physiology maintenance.

## Declarations

### Author contribution statement

Shahran Ahmed Nayem, Nazneen Sultana, Gazi Ziaul Haque Masum: Performed the experiments; Analyzed and interpreted the data; Wrote the paper.

M. Salahuddin M. Chowdhury, Mohammad Abu Hena Mostofa Jamal: Conceived and designed the experiments; Contributed reagents, materials, analysis tools or data; Wrote the paper.

Md. Shahedur Rahman: Analyzed and interpreted the data; Contributed reagents, materials, analysis tools or data; Wrote the paper.

### Funding statement

This work was supported by the Ministry of Science and Technology, Government of the People's Republic of Bangladesh is acknowledged for financial support by providing National Science and Information and Communication Technology (NSICT) fellowship, 2014–15.

### Competing interest statement

The authors declare no conflict of interest.

### Additional information

No additional information is available for this paper.
